# Suicide in Older Prisoners in Germany

**DOI:** 10.3389/fpsyt.2019.00154

**Published:** 2019-03-29

**Authors:** Annette Opitz-Welke, Norbert Konrad, Justus Welke, Katharina Bennefeld-Kersten, Ulrich Gauger, Alexander Voulgaris

**Affiliations:** ^1^Justizvollzugskrankenhaus in der JVA Plötzensee, Berlin, Germany; ^2^Institut fúr Forensische Psychiatrie der Charité Berlin, Berlin, Germany; ^3^Federal Joint Committee, Berlin, Germany; ^4^Institut für Suizidforschung, Reßdorf, Germany; ^5^Institut für Sexualforschung und Forensische Psychiatrie, Universitätsklinikum Hamburg Eppendorf, Hamburg, Germany

**Keywords:** prison, suicide, older prisoners, male prisoners, mental health care, suicide rate

## Abstract

As in many countries, the numbers of older prisoners are rising in Germany, but scientific information on this group is scarce. For the current study, a survey was used that included all prison suicides in Germany between the years of 2000 and 2013. Suicide rates of the elderly prisoners exceeded the suicide rates of the general population and the same age group. We observed a continuous decrease in the suicide rate of elderly prisoners. When compared to the younger suicide victims in prison, significantly more elderly suicide victims were: female, of German nationality, remand prisoners, or serving a life sentence. In Germany, elderly prisoners are a vulnerable subpopulation of the prison population. Higher suicide rates than in the same age group in the general population indicate unmet needs regarding mental disorders and their specific treatment.

## Introduction

For the longest time in history, prison was a matter of the young. Today, in Europe and North America the number of elderly prisoners is rising, although they are still a minority in prison (1). Currently, about 10–16% of the prison population in the western world is over 50 years old and about 3% is over 60 (2). Reasons for the growing number of elderly prisoners are manifold. One unspecific factor is that life expectancy is increasing in general, leading also to aging prison populations (3). Furthermore, older prisoners tend to receive longer sentences often as a consequence of repetitive reoffending (4).

In general, the cut-off age for older persons is 60 years (5), but it has been variously discussed that prison inmates should be considered as old at an earlier age, mostly because analysis of mortality rates revealed that aging is accelerated for persons with a history of incarceration when compared to the general population (6). Therefore, studies on older prisoners have been using different age-groups ranging from 40 to 65 years (3, 6, 7). Comparing frequencies and percentages of age used to denote older inmates, the most common minimum age criterion was 50+ years (8). Findings from an interview study with ex-convicts with a mean age of 55.8 years suggested that this group was characterized by a specific combination of health problems, combining symptoms of post-traumatic stress disorder, social-sensory disorientation, and alienation (9).

In many aspects older prisoners are a vulnerable group. They lack in physical strength compared to their younger inmates what puts them at risk of bullying, harassment, and violence. Due to overcrowding and inadequate resources, they may face more difficulties in getting their specific needs met. In addition, the prison environment of today is not designed to meet the needs of patients at risk for dementia (10). On the other hand, incarceration may actually have health benefits especially for men of the lower or middle class, because life in prison offers regular meals, the possibility to rest often and access to health care services (11, 12). Evaluating data on 87 long-term prisoners over an average period of 14.6 years, Dettbarn was unable to prove a damaging effect of long-term imprisonment (13). Analyzing the cause of death in male prisoners in England and Wales over a period of 20 years, a lower standardized mortality ratio (SMR) was revealed for prisoners compared to the general population and the SMR for the age band 60+ was lower than for the younger age bands (14).

Regarding the prevalence of severe mental disorders, there is evidence that prisoners all over the world are more often mentally ill and that they are affected prematurely by cognitive impairment (15, 16). According to Fazel et al. 10% of male and 14% of female prisoners are diagnosed with severe depression (17). Furthermore, suicide is a leading cause of death in prison (18). Regarding the prevalence of cognitive impairment in older prisoners, a study of Kingston et al. showed that 12% of the examined prisoners aged 50+ years demonstrated signs of cognitive impairment and 50% were diagnosed with a mental disorder. In spite of the high proportion of mentally impaired older prisoners, only 18% received an appropriate, prescribed medication. Therefore, Kingston pointed out that the mental health needs of older prisoners tended to be undetected and untreated. Interviews with prisoners aged 59+ years in England and Wales revealed a high prevalence of depressive disorders, which was five times higher than that found in other studies of younger prisoners and elderly men in the community (19). In an interview study with 124 prisoners aged 50+ years, Barry et al. demonstrated that a past alcohol dependence and a poor self-rated health were associated with elevated suicidal ideation. Altogether, in their study, 22% of the older prisoners were showing current suicidal ideation and 12% were reporting active suicidal ideation (20).

## The Aim of the Study

As in many other countries, the number of older prisoners (age ≥ 50 years) in Germany continuously increased from 2000 to 2013 (21–24). Sound scientific evidence on this matter is scarce. To our knowledge, our study is the first publication on elderly German prisoners who committed suicide. For the first time, a suicide rate of elderly German prisoners will be determined using data of an exhaustive nationwide suicide survey. Our hypotheses are:

1. The suicide rate of elderly prisoners exceeds that of younger ones and suicide rates of younger and elder prisoners exceed the suicide rates of the respective groups in the community.
2. The suicide rates of elderly prisoners are decreasing between the years 2000 to 2013.
3. Some characteristics of the elderly prisoners who committed suicide in prison differ substantially and significantly from the younger prisoners.

## Materials and Methods

Data on all prison suicide events in Germany from the years 2000 to 2013 were collected from a survey using a specific questionnaire on each suicide event in prison. The survey was completed via the reports on exceptional events found in the routine prison documentation and was endorsed by the respective ministries of justice of the German Federal Lands. The respective federal institutions rated the questionnaires, and only 3 Federal Lands were not able to provide the rating of the questionnaires. In these federal lands, the “Generalakten” (Summary files for each prisoner that contain all respective data) were extracted by one of the authors (25). The survey of suicide events in prison comprised a period of 14 years, from January 2000 to December 2013.

The questionnaire that was used for the survey assessed socioeconomic data, data concerning the execution of the sentence and data concerning the course of imprisonment. The items of the questionnaire refer to the history of the prisoner. Since only aggregated data are published, no concern exists for disclosure of personal data. All items, except date of birth, were coded dichotomously. In German prisons, documented medical data is confidential and therefore not part of the “Generalakten.” For that reason, information gained by health professionals is not included. Some variables were assessed incompletely, these are marked by an asterisk.

Sociodemographic data: gender, date of birth, country of birth, nationality, religion^*^, marital status^*^, number of children, housing before incarceration^*^, education^*^, professional qualification^*^, employment before incarceration^*^.

Data on the execution of the sentence and circumstances of the suicide: pre- or post-trial status, number of prior incarcerations, homicide, sexual offenses, actual sentence in month, alcohol/drug involved in actual offense, substance abuse, addiction therapy^*^, remand status, symptoms of alcohol withdrawal^*^, symptoms of drug withdrawal^*^, criminal behavior at a young age, criminal behavior involving close friends or relatives^*^, mental disorder^*^.

Data concerning the course of imprisonment: aggressive behavior, suicidal behavior, physical attacks, social contacts^*^, privileges^*^, escape, disciplinary measures^*^, bullying^*^, date of suicide, cause of death, suicide note, security measures^*^.

In accordance with publications on the elderly prison population, we chose the cut-off age of 50 years for older prisoners in our study (8). For calculating the suicide rates, the number of all subjects imprisoned in the respective years from 2000 to 2013 was taken from the annually published volumes of the German official demographic statistics (26). These reports provided the numbers of prisoners in 5-year age categories (e.g., 20–25, 25–30). Trends in the suicide rates were analyzed by linear regression for the years 2000 to 2013 and age groups (old vs. young) were compared using an ANCOVA-approach. The model we defined uses the suicide rate as dependent and year and age as independent variables. Both suicide rate and year were treated as numeric, while age was coded as 0/1. With that approach, the interaction effect of age and year can be tested. The suicide rates were standardized in order to make them comparable. Subsequently, a linear model was defined in order to test the interaction effect of the age groups and the year. Due to the small number of female prisoners that committed suicide in our sample, we focused the analyses for trends in prison and in the community on male subjects.

Data were analyzed using Pearson's Chi^2^ test for r × c tables. These univariate analyses were done in order to get an overview regarding the possible relationships between variables. In order to identify factors describing the group of the elderly prisoner population, a logistic regression model was developed. Because variables with too many missing cases potentially bias the results, only those variables with at least 60% of valid values were used. The cut-of value of at least 60% valid cases was chosen arbitrary. The model was developed including in a first step all variables as independent predictors for the outcome to belong to the group of elderly prisoners that committed suicide. In a second step, only the statistically significant predictors from step 1 were considered. We presented the *p*-values and the odds-ratios with the confidence intervals. The statistical analyses were conducted with the statistical software R (ver. 3.5.0).

## Results

In the period between the years 2000 to 2013 in total 30 women and 1,037 men died from suicide in German prisons. One hundred seventy-seven men and 11 women (17.6% of all suicide victims) were 50 years of age or older at the time of their death. The suicide rates in prison were consistently higher in the group of older prisoners (age ≥ 50 years). The summary suicide rate from 2000 to 2013 for all age groups was 1,249 per 100,000 prisoners; for younger prisoners (<50) 1,157 per 100,000, for older prisoners 2,042 per 100,000 prisoners.

The suicide rates for men by year and age group were calculated for the prison population as well as for the general population (Table 1).

**Table 1:** Male suicide rates in German prisons and in general population <50 vs. ≥50 years per 100,000 from 2000 to 2013.

	**Male suicide rates per 100,00**			
**Year**	**Male prisoners age < 50 years**	**Male general population age < 50 years**	**Male prisoners age ≥ 50 years**	**Male general population age > 50 years**
2000	159.7	15.0	309.4	31.8
2001	114.0	14.5	239.4	31.6
2002	166.7	14.5	245.8	32.6
2003	131.4	14.2	219.8	32.3
2004	147.0	13.6	240.4	31.6
2005	138.3	12.8	218.3	29.8
2006	111.8	11.8	205.5	29.4
2007	94.0	11.6	211.3	28.0
2008	107.8	11.7	120.3	27.7
2009	103.3	11.8	133.1	28.6
2010	77.6	12.4	280.0	29.0
2011	64.8	12.4	232.7	30.9
2012	107.6	11.9	145.2	28.9
2013	73.9	11.7	115.7	29.7

**Figure 1:**
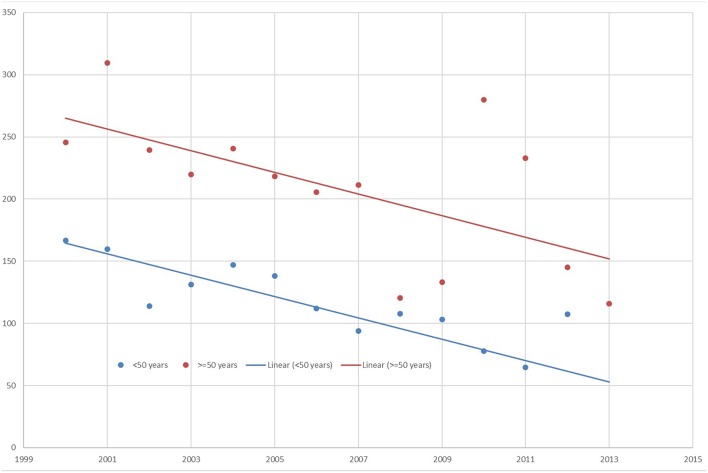
Suicide rates in male prisoners from 2000 to 2013 by age.

A downward trend in the suicide rates in prison applied to both age groups in male prisoners. The downward trends between the group of younger and older prisoners differed not significantly (Interaction year × age 0.13, *t*-statistic 1.75, *p* = 0.09). Parallel to the findings in the prison system, a downward trend in the suicide rates for the general German male population applied to both age groups, but did not differ statistically significantly either (Interaction year × age 0.04, *t*-statistic 0.65, *p* = 0.52). Although the suicide rates of the older prisoners declined continuously from 2000 to 2013, the suicide rates were still higher in comparison to the suicide rates of younger prisoners (Figure 1).

Table 2 shows variables with potential impact on suicidal behavior. Between the younger and older (age ≥ 50 years) suicide victims, there was no statistically significant difference regarding remand status, the perception of high suicide risk by prison personnel, reported bullying preceding the suicide, history of former suicide attempts or special security measures applied. There was a significant difference between the age categories concerning drug withdrawal symptoms, but not for alcohol. Female gender, lifelong sentence, a conviction for crimes against close relatives and sexual offenses were significantly more often positive in the elderly suicide victims. Furthermore, there were statistically significant lower proportions of older suicide victims with a non-German nationality.

**Table 2:** Comparison of characteristics between age groups in German prisoners who committed suicide.

**Variables (*N* valid cases)**	**<50 years: *N* = 876 (% of < 50)**	**≥50 years: *N* = 188 (% of ≥ 50)**	**Test statistic**	***p*-value**
Female (1,067)	19 (2.1)	11 (5.9)	7.71	0.01^**^
Non-German (1,064)	276 (31.5)	30 (16.0)	18.27	<0.001^***^
Remand status (950)	446 (57.8)	105 (58.7)	0.039	0.84
Lifelong sentence (1,067)	25 (2.8)	15 (8.0)	11.32	<0.001^***^
History of suicide attempts (735)	116 (18.9)	21 (17.2)	0.20	0.66
Mental disorder (586)	103 (22.2)	18 (14.6)	3.44	0.64
Bullying (483)	31 (7.9)	6 (6.7)	0.15	0.69
Drug withdrawal (586)	63 (13.6)	2 (1.6)	14.14	<0.001^***^
Alcohol withdrawal (586)	29 (6.3)	9 (7.3)	0.18	0.67
Security measures (605)	105 (22.6)	35 (25.0)	0.091	0.55
Crime involving close relatives (553)	92 (21.1)	54 (46.2)	29.80	<0.001^***^
Sexual offences (1,067)	53 (6)	21 (11.2)	6.34	0.01^*^

* p < 0.05;

** p < 0.01;

*** *p < 0.001*.

To create a regression model, variables with missing data in more than 40% of the cases were excluded. Consequently, the following items were excluded: bullying, mental disorder, crime involving a close relationship, drug, and alcohol withdrawal and security measures. The initial model included 6 variables (Table 3).

**Table 3:** Logistic regression analyses.

	**Estimate**	**Std. error**	**Statistic**	***p*-value**
Gender	−0.91	0.42	−2.15	0.03^*^
Nationality	0.86	0.22	3.86	<0.001^***^
Remand status	0.39	0.18	2.18	0.03^*^
Lifelong sentence	0.95	0.38	2.52	0.01^*^
History of suicide attempts	−0.11	0.20	−0.54	0.59
Sexual offences	0.35	0.21	1.67	0.01^*^

* p < 0.05;

*** *p < 0.001*.

The stepwise removal of variables in the analysis resulted in a model with 4 independent variables: gender, remand status, lifelong sentence, and German nationality (Table 4).

**Table 4:** Variables independently associated with suicide events in older prisoners.

**Variable**	**Odds ratio, 95% Confidence interval**	**p-value**
Male gender	0.44 [0.19; 0.94]	0.05
German nationality	2.45 [1.59; 3.77]	<0.001^***^
Remand status	1.55 [1.09; 2.18]	0.01^*^
Lifelong sentence	2.59 [1.24;5.4]	0.01^*^

* p < 0.05;

*** *p < 0.001*.

The odds ratios indicate that being female, of German nationality, in remand custody or lifelong sentenced as independent predictors to belong to the elderly group.

## Discussion

As hypothesized, between the years 2000 to 2013 the suicide rates of German prisoners aged 50 years and older were higher than the suicide rates of prisoners younger than 50 years. The suicide rates were in general higher in both age groups of prisoners than in the community. We observed a downward trend in both age groups and were able to identify characteristics in the older prisoners who committed suicide in prison that differed statistically significantly from the younger prisoners. The relevant factors that we identified were being female, German nationality, remand custody status, and a lifelong sentence. These factors proved to be independent predictors for belonging in the group of older prisoners that committed suicide.

The finding that the suicide rate of older prisoners exceeds the suicide rate of the younger prisoners is in accordance with results from Donahue et al. who identified older prisoners as a new vulnerable group. According to their findings, elderly prisoners are characterized by a combination of mental and physical health problems and a high rate of vulnerability, and victimization (27). A systematic review of middle-aged and older adults supported a significant association between functional disability and suicidal ideation with depression as a mediator between the two (28).

Kammerer and Spohr interviewed 18 men aged between 65 and 76 and asked them about their situation. The results showed that offers focused on the specific needs of this age group were rated positively, but that further need was apparent (22).

Looking at variables independently in association with age in German prison suicide victims revealed that the items “remand status” and “lifelong sentence” were associated with an increased risk of death by suicide in the group of older prisoners when compared to younger male suicide victims. Higher suicide rates in older remand prisoners may indicate a reduced ability of this age group to cope with imprisonment. In addition, elder men may be disadvantaged regarding physical strength and assertiveness in conflicts with younger inmates, which may result in difficulties to adapt to the prison environment and could lead to a depressive mood and suicidal ideation. Liem and Kunst introduced the idea that older prisoners frequently demonstrate a unique set of mental health problems related to post-traumatic stress disorder. They interviewed ex-prisoners who were released after serving a lifelong sentence and found a specific cluster of mental health problems characterized by institutionalized personality traits, social-sensory disorientation, and alienation. The authors argue that untreated or undertreated mental health problems hinder the successful re-entry into society. Furthermore, these mental health problems may explain the high prevalence of illicit drug abuse, the social withdrawal and (at least partly) the elevated suicide rate (9). According to our findings, Fazel et al. revealed an association between prison suicide events and being sentenced to life in prison in a systematic review (18). Our findings, that older prisoners with a lifelong sentence have a higher risk of committing suicide than their younger counterparts, support the assumption of Turner et al. that older prisoners face a “double burden,” facing a de facto lifelong sentence when incarcerated at an older age (4). Elevated suicide risk in this group may thus be an expression of hopelessness and unmet social needs.

When comparing the groups of younger and older prisoners that committed suicide, we surprisingly observed no differences in the prevalence of mental disorders. The low prevalence of mental disorders in our data set is in stark contrast to findings from medical chart reviews, which report much higher rates of depression and mental disorders in general in older prisoners from 38 to 61% (7, 21, 27). The low prevalence of mental disorders in this group may be a result of the specific difficulties in older prisoners expressing their needs. According to de Smet et al. who studied factors related to the quality of life in older prisoners, psychiatric symptoms seemed to be noted less often in this age group because “older prisoners seem to be poorer self-advocates” than their younger mates (29).

Comparing older German and non-German suicide victims revealed a lower suicide rate in older non-German prisoners. This result is in line with findings from Radeloff et al. who described a significantly higher suicide rate among male German prisoners in comparison to male non-German prisoners (30). This finding contrasts with scientific evidence that immigration is a risk factor for death by suicide (31, 32). Some findings support the idea that a lower suicide risk of immigrants can be explained by descending from a population with a lower suicide risk than the host population (33).

Female prisoners have been described as a vulnerable group before and the suicide rate in this group has increased in Germany from 2000 to 2013 (34, 35). Specific factors connected to the prison setting that contribute to the suicide risk in incarcerated women are so far unknown (36). When compared to male prisoners, the increasing suicide rates in female prisoners in Germany was not linked to a more unfavorable risk profile regarding known risk factors for prison suicide. An analysis of routine data on prisoners who died by suicide in Germany between 2000 and 2013 revealed no significant gender difference regarding most characteristics, especially mean age, nationality, pre- and post-trial status, proportion of individuals serving a life sentence, and proportion of individuals who exhibited criminal behavior at a young age, previous suicide attempts and known history of psychiatric disorder (34).

Although the suicide rate of the prison population was substantially higher than that of the resident population, we found no difference when comparing the trends of suicide rates between the years 2000 to 2013, despite the rising number of older prisoners in the same time span. Factors that contribute to the differences in suicide risk between detainees and the resident population and to the positive trend in male prison suicide rates are not yet understood (37, 38). Although mental disorders, especially depression, are proved to be more common in prisoners than in the general population, the elevated suicide rate among prisoners cannot be explained with individual factors sufficiently (39–41).

## Limitation and Outlook

The main limitations of our study were that there was no control group and that for many items the data was incomplete. Another limitation was that the data source did not include the medical charts so that the prevalence of mental disorders is surpassingly underreported. Future research will include the medical records and should have a case-control design, comparing the cases, who died from suicide, with a matched group of prisoners who survived. The matching variable will be calendar month. From all prisoners admitted in the same month (e.g., September 2005) as the case, one person will be chosen at random as a control.

## Conclusion

In Germany, the number of elderly prisoners is rising. Our findings did not foster the assumption that mental disorders in general or depression are more common among older suicide victims in prison when compared to their younger counterparts. The fact, that hints for mental disorder or suicidal ideations are documented only in few cases, may indicate underreporting of mental disorder in younger as well as in older prisoners. As expected, the suicide rates in male prisoners in Germany with an age of 50 years and older showed a downward trend.

## Ethics Statement

According to current legal regulation, no approval from the local ethics committee was required for the current study. After having signed a formal obligation, the authors were allowed by prison Administration of Lower Saxony to use routine data for research purposes. We attached the respective form that was completed and signed by researchers that participated in the data assessment (see Supplementary Data Sheet 1). The results of the survey are anonymous, name, address before incarceration, and other personal information is not included. Since only aggregated data will be published, no concern exists about disclosure of personal data.

## Author Contributions

AO-W and NK designed the study. KB-K collected the data. AO-W, AV, UG, and JW analyzed and interpreted the data. AO-W and AV wrote the initial draft of the manuscript and had full access to all the data in the study and take responsibility for the integrity of the data and the accuracy of data analysis. All authors have contributed to, read, and approved the final version of the manuscript.

### Conflict of Interest Statement

The authors declare that the research was conducted in the absence of any commercial or financial relationships that could be construed as a potential conflict of interest.

## Supplementary Material

Supplementary Data Sheet 1 Formal obligation.
