# Supplementary material for: Suicide in Older Prisoners in Germany
Source: Front Psychiatry. 2019 Mar 29;10:154. doi: 10.3389/fpsyt.2019.00154 (PMC6449439; doi:10.3389/fpsyt.2019.00154)
Supplement: Supplementary Data Sheet 1 — Formal obligation. [file Data_Sheet_1.PDF]

V E R P F L I C H T U N G S E R K L Ä R U N G

gemäß § 1 des Verpflichtungsgesetzes

Dr. Katharina Bennefeld-Kersten
29478 Restorf……………
Deichstraße 4………………………….

………………………...
Vor- und Zuname des/der zu Verpflichtenden Geburtsdatum

…………………………………………….
Personalausweisnummer

Forschungsvorhaben/Studie/Befragung …Auswertung der Totalerhebung Suiziden von Gefangenen…………………………………………………

Die genannte Person wurde nach Belehrung darauf hingewiesen, dass die von ihr aus Anlass ihres v.g. Forschungsvorhabens/Studie/Befragung vorzunehmende Datenauswertung nur unter Beachtung der folgenden Auflagen stattfinden darf:

1. Die Veröffentlichung der Datenauswertung bedarf der Zustimmung von Frau Bennefeld-Kersten. Es dürfen keine länderspezifischen Daten (z.B. Anzahl der Suizide in NDS) veröffentlicht werden.

2. Die aufgrund der Datenerhebung gewonnenen personenbezogenen Daten dürfen gemäß § 25 NDSG nur für die Forschungsarbeit bearbeitet und genutzt und nur nach Maßgabe der Absätze 3 bis 5 von § 25 NDSG verarbeitet werden.

3. Die personenbezogenen Daten sind gegen unbefugte Kenntnisnahme durch Dritte zu schützen.

4. In der Forschungsarbeit sind die personenbezogenen Daten zu anonymisieren und zwar auch schon auf Fragebögen, in Protokollen oder bei der Erfassung über PC.

Es werden ferner der Inhalt folgender Strafvorschriften des Strafgesetzbuches bekannt gegeben:

§ 133 Abs. 3 Verwahrungsbruch

§	201 Abs. 3	Verletzung der Vertraulichkeit des Wortes
§	203 Abs. 2, 4, 5	Verletzung von Privatgeheimnissen
§	204	Verwertung fremder Geheimnisse
§	353 b	Verletzung des Dienstgeheimnisses

Die benannte Person erklärte sich bereit, sich diesen Vorschriften entsprechend zu verhalten.

………………………………… , den ....

.... ………………………………….

Name, Dienstbezeichnung Verpflichtete/r
